# Gastric carcinogenesis: a comprehensive review of the angiogenic pathways

**DOI:** 10.1007/s12328-020-01295-1

**Published:** 2020-11-18

**Authors:** Alicja Forma, Magdalena Tyczyńska, Paweł Kędzierawski, Klaudyna Gietka, Monika Sitarz

**Affiliations:** 1 Department of Forensic Medicine, Medical University of Lublin, 20-090 Lublin, Poland; 2 Department of Human Anatomy, Medical University of Lublin, 20-090 Lublin, Poland; 3 Department of Conservative Dentistry with Endodontics, Medical University of Lublin, 20-090 Lublin, Poland

**Keywords:** Gastric cancer, Carcinogenesis, Tumorigenesis, Angiogenesis, Pro-angiogenic pathways

## Abstract

Gastric cancer (GC) is undoubtedly one of the most prevalent malignancies worldwide. Since GC is the second leading cause of cancer-related deaths with nearly one million new diagnoses reported every year, there is a need for the development of new, effective treatment strategies of GC. Gastric carcinogenesis is a complex process that is induced by numerous factors and further stimulated by many pro-oncogenic pathways. Angiogenesis is the process of the new blood vessels formation from the already existing ones and it significantly contributes to the progression of gastric tumorigenesis and the growth of the cancerous tissues. The newly formed vessels provide cancer cells with proper nutrition, growth factors, and oxygen supply that are crucial for tumor growth and progression. Tumor-associated vessels differ from the physiological ones both morphologically and functionally. They are usually inefficient and unevenly distributed due to structural transformations. Thus, the development of the angiogenesis inhibitors that possess therapeutic effects has been the main focus of recent studies. Angiogenesis inhibitors mostly affect the vascular endothelial growth factor (VEGF) pathway since it is a major factor that stimulates the pro-angiogenic pathways. The aim of this review was to describe and summarize other promising molecular pathways that might be crucial in further improvements in GC therapies. This article provides an overview of how a meaningful role in tumor progression the angiogenetic process has. Furthermore, this review includes a description of the most important angiogenic factors as well as pathways and their involvement in gastric carcinogenesis.

## Introduction

Gastric cancer (GC) is the fourth most prevalent cancer worldwide and the second most often cause of cancer-related deaths with more than 990,000 new diagnoses and approximately 738,000 death cases reported annually [1, 2]. Males are reported to be nearly two to three times more susceptible to the onset of GC compared to females [3, 4]. The epidemiological studies showed that over 50% of the newly diagnosed patients are from developing countries such as Eastern Europe, East Asia, as well as Central and South America; lower risk areas include Southern Asia, East Africa, and North America [5]. When it comes to the 5-year survival rate, only Japan achieved mildly good results, whereas the European ratio oscillates between 10 and 30% [6]. Throughout the last few decades, it has been observed that GC incidence rates decreased in most parts of the world; however, this data subject only to the sporadic intestinal type of GC; the prevalence of diffuse GC has increased [7]. Currently, more cases of the proximal GC than the distal one are observed among patients [8]. Numerous risk factors contribute to the onset of GC, among which *Helicobacter pylori* (*H. pylori*) infection, genetic factors, imbalanced diet, as well as excessive alcohol consumption, are of the highest importance [9–14].

Currently, two major histological classifications of GC are distinguished—the most commonly used—the Lauren classification and the most detailed one—the World Health Organization (WHO) classification [15]. Besides, another division that concerns the patient’s age was proposed—an early-onset gastric carcinoma is mostly identified in patients aged 45 or younger, whereas, the conventional GC—in patients older than 45 [16, 17]. In addition, another classification based on the next-generation sequencing (NGS) was proposed and it includes four major molecular subtypes—MSI, MSS/TP53^+^, MSS/TP53^−^, and MSS/EMT [18].

Gastric carcinogenesis is a complex, multifactorial process that is primarily stimulated by chronic inflammation (induced by *H. pylori* infection in the majority of cases); however, other processes such as the epithelial–mesenchymal transition (EMT) or intensified angiogenesis play a crucial role in GC progression [19–23]. Angiogenesis is a physiological process of the formation of the new blood vessels from the already existing ones. Apart from its relevance under the physiological conditions, angiogenesis is also crucial for the growth of the cancerous tissues, as the newly formed vessels provide nutrition, growth factors, and oxygen supply [24, 25]. Under physiological conditions, angiogenesis is mainly a consequence of the hypoxic and ischemic signals, whereas, in the pathological conditions, angiogenesis is uncontrolled and upregulated due to the predominance of the pro-angiogenic factors [26, 27]. Pathological angiogenesis is characterized by the atypical morphology of the tumor vasculature, as well as the increased proliferation of the endothelial cells (ECs), pericytes, smooth muscle cells, and the basement membrane [28]. Tumor-associated vessels differ from the normal vessels both morphologically and functionally. Physiological vessels are usually evenly distributed and due to their effective coating by the pericytes, they are able to provide efficient delivery of the nutrients and oxygen. Contrarily, tumor vessels are often inefficient because of the structural transformations–they tend to be unevenly distributed and form tortuous and overall chaotic networks with an irregular branching. Moreover, tumor vasculature is not perfused regularly and it presents a bidirectional blood flow [29, 30]. The progress and development of the cancerous tissues and their metastases result from an efficient vascular response. The process starts with an “angiogenic switch” that enables tumor growth and expansion beyond the primary site of tumorigenesis. The switch consists of the following steps: perivascular detachment and vessel dilation, angiogenic sprouting, creation of the new blood vessels, as well as the recruitment of the perivascular cells [31, 32]. Those processes can occur during every stage of the carcinogenesis—before, during, or even after tumor progression [33, 34].

Treatment strategies of GC include surgery, radiation, therapy, chemoradiation, chemotherapy, and targeted therapy. Some research demonstrated that metformin might also constitute a potential treatment for several types of cancer including GC [35–37]. Other potential strategies for the advanced GC include the decrease of the pro-angiogenic ligands levels and the expression of their receptors, the increase of the angiogenic inhibitors levels, as well as directly targeting the inner walls of the ECs [38]. Furthermore, the inhibition of the angiogenesis indirectly enhances the effects of chemotherapy, most likely because of the vascular normalization and more effective delivery of the chemotherapeutic agents (Table 1) [39–41]. The purpose of this review is to accentuate and evaluate the significance of the pro-angiogenic pathways in GC patients. Furthermore, we aimed to summarize the most meaningful pro-angiogenic factors and their receptors, as well as potential molecular pathways and their involvement in gastric carcinogenesis.

**Table 1:** The anti-angiogenic drugs that might be used in GC patients

Anti-angiogenic drug	Biological category/mechanism of action	Current development status*	Clinical indications
Bevacizumab	Ani-VEGF human monoclonal antibody (IgG1)	Phase IV	Gastric cancer, colorectal cancer, non-small cell lung carcinoma, breast cancer, renal cell cancer, glioblastoma multiforme, diabetic retinopathy, macular degeneration, ovarian cancer
Ramucirumab	Anti-VEGFR-2 human monoclonal antibody (IgG1)	Phase IV	Gastric cancer, gastro-esophageal junction adenocarcinoma, colorectal cancer
Sunitinib	Tyrosine kinase inhibitor	Phase II	Gastric cancer, gastrointestinal stromal cancer, meningioma, renal cell carcinoma, breast cancer, non-small cell lung carcinoma, neuroendocrine tumors, leukemia
Sorafenib	Tyrosine kinase inhibitor	Phase II	Gastric cancer, renal cell carcinoma, hepatocellular carcinoma, thyroid cancer, desmoid tumors
Apatinib	Tyrosine kinase inhibitor	Phase IV	Gastric cancer, breast cancer, hepatocellular carcinoma
Aflibercept	VEGF-Trap; targets VEGF-A, VEGF-B, and PIGF	Phase II	Gastric cancer, macular degeneration, colorectal cancer
Cetuximab	EGFR inhibitor	Phase III	Gastric cancer, squamous cell carcinoma

*For GC patients

## Biomarkers of angiogenesis

The carcinogenesis is associated with the release of the specific biomarkers that can be used for diagnosis, prognosis, and even selection of the proper treatment therapy. In GC, tumor cells release large amounts of the molecules that induce the growth of the new blood vessels, promoting the angiogenesis process. Studies have shown that GC cells present a high angiogenic potential by secreting the pro-angiogenic cytokines (such as angiopoietin-1, -2, -3, -4, and tryptase) that stimulate the ECs and the stromal cells, as well as the autocrine loop [42]. Specific biomarkers of angiogenesis might be crucial in assessing a patient’s response to the particular anti-angiogenic therapy and predicting the overall clinical outcome of patients. Currently, the most described and best-known biomarkers of angiogenesis include the vascular endothelial growth factor and epidermal growth factor families, placental growth factor (PIGF), resistin-like-molecule-α (RELM-α), angiopoietins, platelet-derived growth factor-β (PDGF-β), fibroblast growth factor (FGF), hypoxia-inducible factor (HIF), tryptase, integrins, and IL-8.

### Vascular endothelial growth factor family

Vascular endothelial growth factors (VEGFs) constitute a group of polypeptides that are considered to be one of the most crucial factors involved in the angiogenesis processes, which expression is significantly increased during gastric carcinogenesis [43]. Some of the members of the VEGF family are not only the pro-angiogenic factors crucial in the pathogenesis of the angiogenesis-related diseases, but play a role in the formation of the lymphatic vessels as well [44]. The VEGF family consists of the 7 major subtypes including VEGF-A, VEGF-B, VEGF-C, VEGF-D, VEGF-E, VEGF-F, and PlGF [45]. VEGF family signaling is mediated by binding to the tyrosine kinase receptor (VEGFR-1, -2, -3), wherein VEGFR-2 is considered to be the main angiogenesis mediator. Vascular endothelial growth factor A (VEGF-A) is the first described cytokine that is considered to be the primary survival factor of the vascular ECs; VEGF-A stimulates their proliferation and migration, modulates the permeability, and inhibits the apoptosis [24, 46, 47]. VEGF-A, which is released in the excessive amounts from the tumour cells, is now considered as the strongest pro-angiogenic factor in gastric tumorigenesis [48, 49]. The expression of VEGF-A might increase the density and number of vessels in the intestinal as well as the diffuse-type of GC [50]. VEGF-A regulates vascular permeability and gene expression by the attachment and activation of the VEGFR-1 and VEGFR-2 [51]. Other VEGFs are also involved in the regulation of the angiogenesis and might be expressed either during embryogenesis or during adult stages usually under stressed or pathological conditions. Besides, Maeda et al. showed that patients with VEGF-positive tumours had a poorer prognosis compared to patients with VEGF-negative tumours [52].

### Vascular endothelial growth factor receptors

VEGF receptor 1 (VEGFR1), VEGF receptor 2 (VEGFR2), and VEGF receptor 3 (VEGFR3) are tyrosine kinase receptors that constitute a site of attachment for the members of the VEGF family. These receptors are highly expressed on the ECs and allow VEGF family proteins to be released into the serum [43]. Except for angiogenesis, VEGFRs are also crucial in the infiltration of the immune cells into the tumor microenvironment, promoting persistent inflammation [53]. The binding of VEGFs to their specific receptors promotes angiogenesis by either the production of the signalling intermediates such as phosphatidyl inositol-3-kinase (PI3K), AKT, phospholipase C-γ (PlC-γ), and small GTPases or by the induction of the pro-angiogenic signaling pathways including the PLCγ-PKC-MAPK pathway initiated by the VEGFR-2 [54, 55]. VEGF/VEGFR interactions also stimulate the mitogenesis and cellular migration as well as the recruitment and proliferation of the ECs.

### Epidermal growth factor family

Epidermal growth factor (EGF) is a ligand that binds to the tyrosine kinase receptor (EGFR); its overexpression in solid tumours is associated with an enhanced progression of carcinogenesis and a poorer clinical outcome of patients. EGF/EGFR is involved in the pro-angiogenic processes by regulating growth and maturation of the pathological vessels, stimulating the levels of VEGFR mRNA, as well as the enhanced neuropilin-1 (a coreceptor of VEGFR-2) release [54]. EGF activity is associated with the increased proliferation of the ECs, angiogenesis, as well as reduced apoptosis. The pro-angiogenic activity of EGF is facilitated due to the induction of several pro-oncogenic signalling pathways such as the RAS-RAF-MEK-ERK MAPK and AKT-PI3K-mTOR pathways [56, 57]. Except for angiogenesis, some of the signalling pathways such as TGF*α*-EGFR, stimulate the formation of the lymphatic vessels and metastasis [55].

### Placental growth factor

Placental grow factor (PIGF), a member of the VEGF family, controls the trophoblast growth and differentiation, and it has recently gained interest in carcinogenesis, because of its possible involvement in the angiogenic processes of several solid tumors and leukemia [58–61]. PIGF is mainly overexpressed during embryogenesis and stimulates angiogenesis and vasculogenesis; however, its overexpression during adulthood might induce pathological pro-angiogenic processes during carcinogenesis. PIGF presents a high affinity to VEGFR-1. Researchers demonstrated that among patients with GC, PlGF expression is highly associated with the metastases to lymph nodes, tumour stages, and poor overall survival [61, 62]. The upregulation of the PIGF levels affects the VEGF/VEGFR pathway that further stimulates the pro-angiogenic pathways, the proliferation of ECs, as well as the enhanced inflammation.

### Resistin-like molecule-α

Resistin-like molecule-α (RELM-α) is a marker of the anti-inflammatory macrophages and is highly related to the GC progression. Chen et al. showed that RELM-α expression is associated with the size of the tumour and its advanced stage [63]. The researchers also found that RELM-α activates VEGF by activating the NF-κB-MMP-9/VEGF pathway that facilitates angiogenesis. Besides, RELM-α increases the expression of the other pro-angiogenic factors such as VEGF or vascular endothelial cell adhesion molecule-1 (VCAM-1) [64]. RELM-α is now considered as a novel biomarker of GC which inhibition might potentially reduce the progression and invasiveness of GC.

### Angiopoietins

Angiopoietins (Angs) constitute a family of the angiogenic biomarkers of GC that belong to the family of vascular growth factors and are involved in the control of the embryonic as well as postnatal angiogenesis processes. Angs (Ang-1, -2, -3, -4) bind to the tyrosine kinase receptor tie-2 and are crucial for vessel maturation, migration, adhesion, and the survival of ECs. Ang-2 which disrupts the connections between the perivascular cells and the endothelium promotes cell death with vascular regression and that results in its critical role in tumor angiogenesis [65–67]. Angiopoietin-1 (Ang-1) and angiopoietin-2 (Ang-2) are currently the most examined and are known to be firmly expressed in GC [43, 68]. Both Angs are ligands for Tie-2. By activating Tie-2, Ang-1 stabilizes the vessels by recruiting pericytes [69]. Contrarily, Ang-2 antagonizes Tie-2, thus prevents Ang-1 maturation and has an impact on the growth and maturation of the vessels [70, 71]. Imbalances between Ang-1 and Ang-2 levels might occur independently of each other which is further associated with the severity of the pro-angiogenic processes. The angiopoietin/Tie-cascade is one of the signalling pathways that is involved in the regulation of tumor angiogenesis.

### Platelet-derived growth factor-β

Platelet-derived growth factor-β (PDGF-β) belongs to the PDGF family that binds to the PDGFR-β homodimer. PDGF-β plays an important role in the progression of tumours as they can induce both—the angiogenesis and EMT—that are crucial in tumorigenesis [72–74]. Moreover, Suzuki et al. found that PDGF-β and VEGF have been secreted simultaneously in gastric tumours; however, PDGF-β was more crucial than VEGF in the maintenance of the vessels in the intestinal type of GC [75]. PDGF-β is highly expressed by the ECs and also promotes the recruitment of the perivascular cells. Besides, PDGF-β plays a role in the migration, proliferation, and adhesion of the endothelial progenitor cells, as well as the increased VEGF expression within the tumor microenvironment, that are crucial for both neovascularization and re-endothelialization [76].

### Fibroblast growth factors

Fibroblast growth factors (FGFs) constitute a family of ligands that stimulate and regulate neovascularization and revascularization. FGF binds to four different receptors (FGFR -1, -2, -3, and -4) and regulates cell proliferation, migration, and survival. FGF-1 and FGF-2, by the activation of the AKT pathway, are considered to be one of the most important angiogenesis controllers [77, 78]. There is an association between the activity of FGFs, VEGFs, and inflammatory cytokines, and this synergy significantly facilitates the vascularization, enhancing tumor growth and progression [79]. FGFs facilitate the proliferation of the hypoxic ECs, initiating inflammation; besides, FGFs initiate the ‘angiogenic phenotype’ of the ECs and stimulate them to release a urokinase-type plasminogen activator with a pro-angiogenic activity [80]. Moreover, FGFs are reported to be the potential survival factors of the vascular cells [81]. It was demonstrated that the interaction between FGFs and pentraxin 3 (PTX3) might be related to angiogenesis during the growth of the cancerous tissues [82].

### Hypoxia-inducible factors

Since the hypoxic conditions act as a main driving force for the angiogenesis, the pro-angiogenic activity within the tumor microenvironment is also regulated by the hypoxia-inducible factors 1 and 2 (HIF-1 and HIF-2). The two isoforms of HIF—HIF-1α and HIF-2α—are mainly involved in the pro-angiogenic processes during tumorigenesis. The major role of HIF-1 is to monitor the cellular response to the oxygen levels in solid tumors, whereas HIF-2 is primarily involved in the regulation of the erythropoietin levels [83, 84]. HIF induces the expression of numerous pro-angiogenic factors including VEGFs, PDGF-β, plasminogen activator-inhibitor-1(PAI-1), and Angs (1 and 2), as well as regulates the remodeling of the pathophysiological vessels [85]. Besides, the overexpression of HIF-1α is associated with the mutations of the several oncogenes (p54, PTEN, or VHL) and thus, HIF-1α overexpression significantly affects the tumour growth, metastasis, and invasion properties [86]. HIF stimulates physiological neovascularization under the hypoxic conditions; however, its overexpression leads to the pathological pro-angiogenic processes, which for example is observed as excessive recruitment, stimulation, and proliferation of ECs, or the regulation of the pro-angiogenic genes expression [87].

### Tryptase

Apart from the above-mentioned and well-known pro-angiogenic activators, recent research showed that tryptase, a non-classical moderator, can stimulate angiogenesis both in vitro and in vivo. Tryptase stimulates ECs proliferation and activation of the proteinase-activated receptor-2 (PAR-2) with VEGF as a final product of this process [88, 89]. Tryptase is also involved in the overactivation of the endothelial progenitor cells via AKT and ERK signalling pathways [90]. Moreover, tryptase presents an ability to alter the composition of the extracellular matrix which facilitates angiogenesis [91]. The presence of the tryptase-positive mast cells within the tumor microenvironment is crucial in the initiation of the pathological angiogenesis [92, 93]. Tryptase-positive mast cells activate the c-Kit receptor pathway that further stimulates the gastric carcinogenesis [94]. Thus, tryptase affects angiogenesis either directly or indirectly and might constitute a potential biomarker of the angiogenesis in several types of cancers including GC.

### Integrins

Other molecules—integrins—significantly affect the interactions between the tumor and stromal tissues [95]. The integrins involved in the tumor angiogenesis include several heterodimers such as α1β1, α2β1, α4β1, α5β1, α6β1, α6β4, α9β1, αvβ3, and αvβ5. Integrins play a pivotal role in the regulation of the ECs migration and survival, as well as the enhanced recruitment of the monocytes to the tumor microenvironment [96]. Tumor angiogenesis is also facilitated by the stimulation of the VEGF pathways by integrins and the integrins-related reorganization of the extracellular matrix promoting the microenvironment that is more prone to the pro-angiogenic alterations. Except for angiogenesis, integrins are also involved in the regulation of the lymphangiogenesis and both of these processes significantly affect the tumor metastasis to other organs, worsening the clinical outcome of patients [97]. Therefore, it was proposed that integrins might act as potential biomarkers of angiogenesis and their inhibition alone or combined with other treatment strategies might be crucial during GC therapy.

### Interleukin-8

Interleukin-8 (IL-8) is one of the pro-angiogenic chemokines released by the tumor-infiltrating macrophages involved in the regulation of angiogenesis in various types of cancers. The pro-angiogenic effects of the IL-8 action are stimulated by both—the paracrine and autocrine routes in carcinogenesis. IL-8 induces the overexpression of the VEGF-A, VEGFR-1, and VEGFR-2 suggesting that it might act as a potential biomarker of angiogenesis in GC patients [98]. IL-8 also stimulates the proliferation, survival, and migration of the ECs via the Src/Vav2/Rac1/PAK1 signaling pathway [99]. Besides, IL-8 is involved in the regulation of the capillary tube organization [100]. IL-8 is believed to be a promising marker of several gastrointestinal cancers including GC or colorectal cancer.

## Angiogenic pathways in gastric cancer

**Fig. 1:**
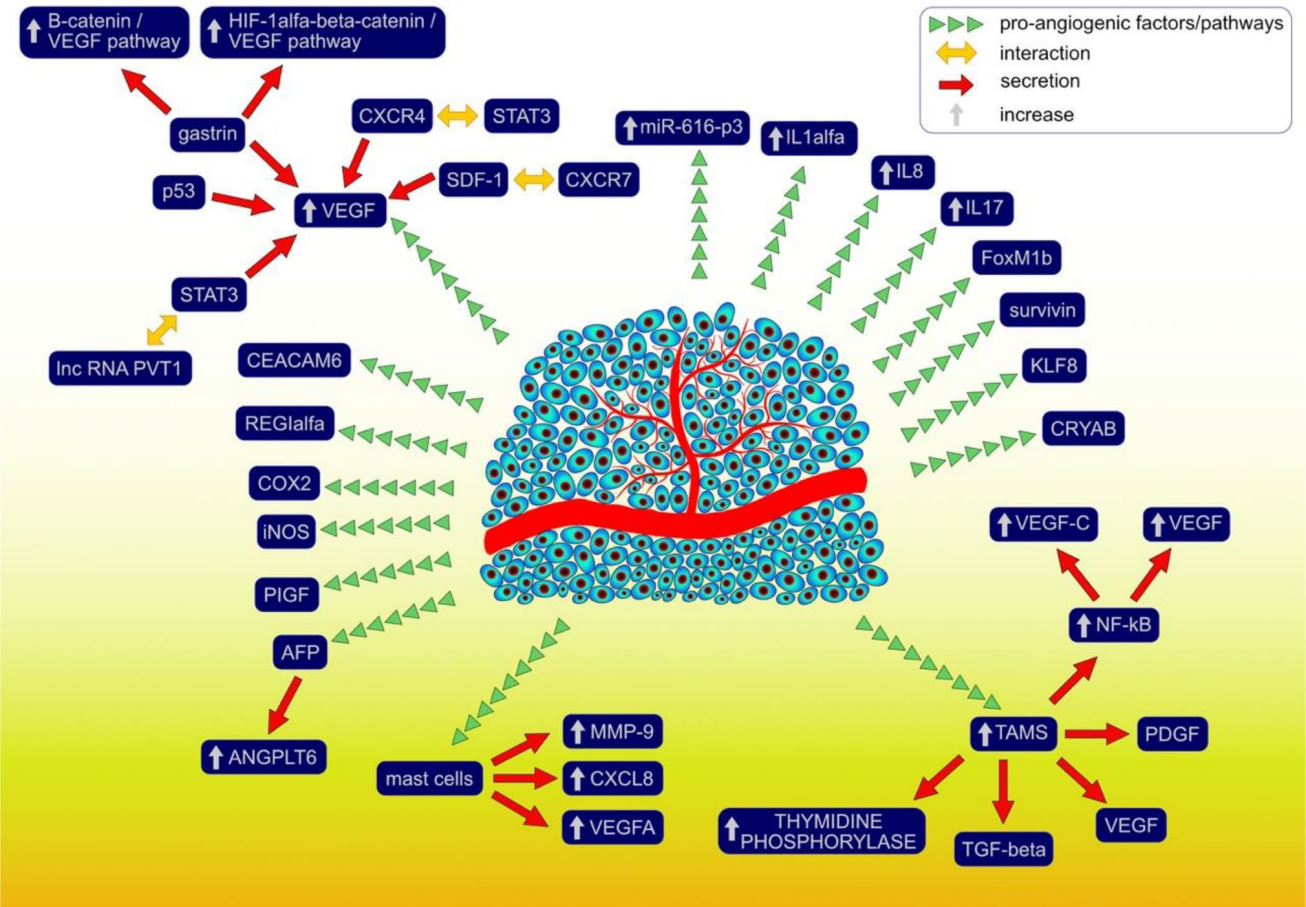
Molecular factors and pathways that are involved in the angiogenesis process during gastric carcinogenesis

Angiogenesis is a complex process that is continually stimulated by the numerous pro-angiogenic and anti-angiogenic factors, as well as molecular pathways enabling further progression of gastric carcinogenesis, growth of the cancerous tissues, and metastasis (Fig. 1). So far, a great number of the pro-angiogenic factors that contribute to gastric carcinogenesis have been described. VEGFR-2, activated by the VEGF attachment, facilitates the formation of the new blood vessels providing oxygen supply and essential nutrients for the gastric cancerous tissues. Moreover, VEGFR-2 also regulates survival, progression, and invasion of GC in a VEGF-independent manner [101]. It was demonstrated that the interaction between stromal cell-derived factor-1 (SDF-1) and the C-X-C motif chemokine receptor (CXCR7) induces the secretion of the significant amounts of VEGF [102]. Besides, the knockdown of CXCR7 prevents further VEGF release and thus, it might constitute one of the potential molecular targets preventing the progression of angiogenesis. Except for angiogenesis, the SDF-1/CXCR7 pathway is also involved in the proliferation, adhesion, and invasion of the tumor cells. Another C-X-C motif chemokine receptor—CXCR4—promotes gastric carcinogenesis via a signal transducer and the activator of transcription 3 (STAT-3)-dependent upregulation of the VEGF levels [103]. Gastrin enhances angiogenesis in either normoxic or hypoxic conditions eventually upregulating the VEGF levels; under normoxic conditions, gastrin promotes the activation of the β-catenin/VEGF pathway, whereas, under the hypoxic conditions, HIF-1α/β-catenin/VEGF pathway is overactivated [104]. HIF-1α regulates the expression of the pro-angiogenic genes such as *VEGF*, significantly contributing to the regulation of the angiogenesis process. VEGFA expression is also upregulated by the interaction between lncRNA PVT1 and the STAT3 pathway; except for the pro-angiogenic effects, the oncogenic effects are further enhanced by the continuous stimulation of PV1 transcription by the activated STAT3 pathway [105]. Besides, aberrant expression of one of the tumor suppressor genes—p53, might promote angiogenesis via the upregulation of the VEGF levels at the same time decreasing the amounts of thrombospondin-1 that acts as angiogenesis inhibitor [106]. The abovementioned finding is of high importance during tumorigenesis since the majority of cancers are characterized by either the loss of or dysregulated p53 functions; p53 is the most frequently mutated gene. VEGF expression can also be increased by the urokinase plasminogen activating system (PA system) contributing to the pro-angiogenic activities and further tumor progression and invasion [107].

Tumor-associated macrophages (TAMs) that have an M2-like phenotype have an ability to induce angiogenesis and promote the proliferation of the cancer cells within the gastric microenvironment. Except for angiogenesis, TAMs also stimulate lymphangiogenesis and those two processes might occur simultaneously. TAMs induce the release of numerous pro-angiogenic factors including TGF- β, VEGF, PDGF, as well as angiogenic chemokines [108, 109]. TAMs promote NF-kB activation and further overexpression of the VEGF and VEGF-C levels promoting angiogenesis and lymphangiogenesis in the GC tissues [110]. Moreover, there is a correlation between patients’ serum VEGF and VEGF-C levels and the number of TAMs observed within the cancerous specimens. Park et al. (2015) showed that CD163 + TAMs promote angiogenesis and induce greater CXCL12 expression in GC patients [111]. The overexpression of CXCL12 stimulates further recruitment of TAMs into the gastric microenvironment inducing tumor cell invasion and progression of gastric carcinogenesis. Besides, TAMs that are characterized by the overexpression of the thymidine phosphorylase (TM) (an angiogenesis-promoting enzyme) levels, are associated with greater tumor angiogenesis and worse clinical outcome and survival of patients with the intestinal type of GC [112]. Other cells that contribute to the progression of angiogenesis include mast cells releasing great amounts of the pro-angiogenic factors such as VEGF-A, CXCL8, or MMP-9 [113]. Except for angiogenesis, mast cells are also involved in the lymphangiogenesis during GC progression. There is a correlation between the extent of angiogenesis as well as malignancy grade and the amounts of the mast cells infiltrating the cancerous tissues. Ribatti et al. (2010) showed that the density of the tryptase-positive mast cells was significantly greater than chymase-positive mast cells in the examined GC specimens; however, it should be considered that all of the mast cells express tryptase activity [114]. Tryptase, as MMP-9, degrades gelatin, thus, facilitates the angiogenesis. Nevertheless, the greater cumulation of the chymase-positive cells can also promote angiogenesis providing further tumor growth primarily by the MMP-9 activation [115].

The expression of cyclooxygenase-2 (COX-2) that is usually highly abundant in GC tissues correlates with the enhanced angiogenesis also contributing to the greater proliferation of GC cells and lymph node metastasis [116]. COX-2 expression also promotes the apoptosis of the GC cells affecting the overall outcome [117]. COX-2 stimulates angiogenesis primarily by modulating the release of VEGF and basic fibroblast growth factor [118]. It was shown that COX-2 inhibitors might significantly reduce angiogenesis, at the same time suppressing the proliferation of the cancer cells and weakening the invasiveness [119, 120]. COX-2 as well as nitric oxide synthase (iNOS), except for stimulating neovascularization and cancer progression, can also act as potential biomarkers indicating the survival of GC patients [121, 122].

Regulation and progression of angiogenesis are also controlled by the regenerating gene (REG) Iα that also stimulates the growth of the cancerous tissues and the anti-apoptotic effects on the endothelial cells [123]. GCs that produce high amounts of α-fetoprotein (AFP) are more prone to induce rich neovascularization, high proliferative activity, and weak apoptosis compared to AFP-negative GCs [124]. AFP-positive cancers are considered to be associated with poorer clinical outcome and prognosis of GC patients. Besides, AFP-positive cancers express high levels of ANGPTL6 that additionally promotes tube formation and ECs migration via the activation of the ERK1/2 and AKT pathways [125]. GC metastasis and angiogenesis can also be enhanced by the overexpression of the carcinoembryonic antigen-related cell adhesion molecule 6 (CEACAM6) and further induction of the FAK signaling pathway [126]. The expression of the PIGF, a member of the VEGF family, is also associated with greater vascularization, lymph node metastasis, serosal invasion, and the general clinical outcome of patients [127]. Other factors that might promote gastric angiogenesis either directly or indirectly include survivin, the mammalian Forkhead Box (Fox) transcription factor FoxM1b, Krüppel-like factor 8 (KLF8), or alpha-B crystallin (CRYAB) [128–131]. The inactivation of the *PTEN* gene might also contribute to the enhanced vascularization process [132]. The regulation of several pro-angiogenic factors such as VE-cadherin, MMPs-2 and 9, MT1-MMP, p-ERK, β-catenin, 23 p-FAK, or p-paxillin was reported to be controlled by an oncogenic long non-coding RNA—MALAT1—which overexpression correlates with poor clinical outcome of GC patients [133]. Angiogenesis is also regulated and stimulated by a vast number of interleukins and their receptors among which IL-1α, IL-8 (via high-mobility group box-1 (HMGB1) contribution), IL-17 (through STAT3 pathway), or soluble interleukin-2 receptor (sIL-2R) are considered to be of the highest importance regarding GC patients; except for neovascularization, IL-1α is involved in liver metastasis of GC [134–137].

The dysregulation of microRNAs (miRs) has also been demonstrated to play a significant role in the induction and progression of angiogenesis in GC patients. GC tissues express the upregulated levels of miR-616-3p that significantly contribute to the induction and progression of angiogenesis as well as the EMT process primarily via the activation of the PTEN/AKT/mTOR pathway [138]. MiR-130a and miR-495 downregulate the Runt-related transcription factor 3 (RUNX3) expression ultimately enhancing angiogenesis and gastric cell proliferation [139]. VEGF-A is primarily targeted by miR-125a and miR-126, facilitating vascularization; other mechanisms of miR-related angiogenesis include the forkhead box O1 (FOXO1) inhibition by miR-135b [140–142]. Contrarily, enhanced expression of miR-218 contributes to the inhibition of the tumor growth by preventing vascularization via targeting ROBO1 [143].

## Conclusions

Angiogenesis constitutes a crucial component of a complex gastric carcinogenesis process enabling quicker and more effective growth of the cancerous tissues. The understanding of the mechanisms that drive angiogenesis during gastric carcinogenesis constitutes a basic approach to provide potential anti-angiogenic therapies. The majority of the currently available anti-angiogenic therapies for GC patients include those that primarily focus on the VEGF-VEGFR pathway specifically. Besides, the pro-angiogenic factors described in this review differ in terms of their clinical significance and application; in GC patients, the expression of VEGFR-2, CXCR7, EGF, VEGF, IL-8, miRNAs (such as miR-218, miR-135b, miR-495, or miR-130a), or TAMs proliferation seem to be associated with the survival rates, thus, they might act as potential prognostic factors of GC. The molecular components and pathways described in this review provide an insight into a vast number of the pro-angiogenic mechanisms that should be further studied to improve the available treatment strategies. The factors and pathways described in this review might also constitute potential molecular targets for establishing new effective anti-angiogenic therapies for GC patients.

## Abbreviations

AFP: α-Fetoprotein
Angs: Angiopoietins
CEACAM6: Carcinoembryonic antigen-related cell adhesion molecule 6
COX-2: Cyclooxygenase-2
CRYAB: Alpha-B crystallin
CXCR4: C-X-C motif chemokine receptor 4
CXCR7: C-X-C motif chemokine receptor 7
ECs: Endothelial cells
EGF: Epidermal growth factor
EMT: Epithelial–mesenchymal transition
FGFs: Fibroblast growth factors
Fox: Mammalian forkhead box
FOXO1: Forkhead box O1
GC: Gastric cancer
HIF-1 and HIF-2: Hypoxia-inducible factor 1 and 2
HMGB1: High-mobility group box-1
iNOS: Nitric oxide synthase
KLF8: Krüppel-like factor 8
miRs: MicroRNAs
mTOR: Mammalian target of rapamycin
NGS: Next-generation sequencing
PA system: Urokinase plasminogen activating system
PAR-2: Proteinase-activated receptor-2
PDGF-β: Platelet-derived growth factor-B
PI3K: Phosphatidyl inositol-3-kinase
PlC-γ: Phospholipase C-γ
PIGF: Placental growth factor
PTX3: Pentraxin 3
RELM-α: Resistin-like-molecule-alfa
RUNX3: Runt-related transcription factor 3
SDF-1: Stromal cell-derived factor-1
STAT-3: Signal transducer and activator of transcription 3
TAMs: Tumor-associated macrophages
TM: Thymidine phosphorylase
VCAM-1: Vascular endothelial cell adhesion molecule-1
VEGF: Vascular endothelial growth factor
VEGF-A: Vascular endothelial growth factor A
VEGFR2: Vascular endothelial growth factor receptor 2
WHO: World Health Organization
